# A tree-like Bayesian structure learning algorithm for small-sample datasets from complex biological model systems

**DOI:** 10.1186/s12918-015-0194-7

**Published:** 2015-08-28

**Authors:** Weiwei Yin, Swetha Garimalla, Alberto Moreno, Mary R. Galinski, Mark P. Styczynski

**Affiliations:** Key Laboratory for Biomedical Engineering of Education Ministry, Department of Biomedical Engineering, Zhejiang University, Hangzhou, P. R. China; School of Chemical & Biomolecular Engineering, Georgia Institute of Technology, 311 Ferst Drive NW, Atlanta, GA 30332-0100 USA; School of Biology, Georgia Institute of Technology, Atlanta, GA USA; Division of Infectious Diseases, Emory Vaccine Center, Yerkes National Primate Research Center, Emory University School of Medicine, Emory University, Atlanta, GA USA

**Keywords:** Bayesian networks, Network learning algorithm, Tree-like networks, Malaria, Non-human primate, Transcriptomics

## Abstract

**Background:**

There are increasing efforts to bring high-throughput systems biology techniques to bear on complex animal model systems, often with a goal of learning about underlying regulatory network structures (e.g., gene regulatory networks). However, complex animal model systems typically have significant limitations on cohort sizes, number of samples, and the ability to perform follow-up and validation experiments. These constraints are particularly problematic for many current network learning approaches, which require large numbers of samples and may predict many more regulatory relationships than actually exist.

**Results:**

Here, we test the idea that by leveraging the accuracy and efficiency of classifiers, we can construct high-quality networks that capture important interactions between variables in datasets with few samples. We start from a previously-developed tree-like Bayesian classifier and generalize its network learning approach to allow for arbitrary depth and complexity of tree-like networks. Using four diverse sample networks, we demonstrate that this approach performs consistently better at low sample sizes than the Sparse Candidate Algorithm, a representative approach for comparison because it is known to generate Bayesian networks with high positive predictive value. We develop and demonstrate a resampling-based approach to enable the identification of a viable root for the learned tree-like network, important for cases where the root of a network is not known *a priori*. We also develop and demonstrate an integrated resampling-based approach to the reduction of variable space for the learning of the network. Finally, we demonstrate the utility of this approach via the analysis of a transcriptional dataset of a malaria challenge in a non-human primate model system, *Macaca mulatta*, suggesting the potential to capture indicators of the earliest stages of cellular differentiation during leukopoiesis.

**Conclusions:**

We demonstrate that by starting from effective and efficient approaches for creating classifiers, we can identify interesting tree-like network structures with significant ability to capture the relationships in the training data. This approach represents a promising strategy for inferring networks with high positive predictive value under the constraint of small numbers of samples, meeting a need that will only continue to grow as more high-throughput studies are applied to complex model systems.

**Electronic supplementary material:**

The online version of this article (doi:10.1186/s12918-015-0194-7) contains supplementary material, which is available to authorized users.

## Background

While systems biology techniques—whether experimental or computational – are often developed on simple model systems, their application to increasingly complex model systems is one of the most exciting and promising aspects of modern biological research. However, applying these techniques to complex systems often presents new challenges. For example, systems biology approaches are only recently being brought to bear on non-human primate model systems [1–3], which can be critical to translational biomedical research when simpler organisms are not good models of human physiology [4]. However, the number of experimental samples possible in these systems is limited: using a large cohort is cost-prohibitive and ethically questionable, and animal welfare considerations limit the volume and frequency of blood or other tissue sampling. Also, validation experiments in non-human primates are extremely difficult, which makes it critical that only a small number of high-confidence hypotheses are tested.

Learning regulatory networks is a common task in systems biology research [5, 6], and one that is confounded by the restrictions associated with complex model systems. Complex model systems usually do not allow for a large number of samples, but robustly learning network structure with few samples is difficult [7, 8]. For experimental validation complex model systems require identification of only a few high-confidence connections between variables, but many common network analysis tools instead generate high-connectivity graphs [9] (due to indirect effects).

Given large sample sizes, Bayesian networks are effective at identifying a small number of meaningful connections between features. Bayesian networks [10] are probabilistic graphical models that account for conditional dependencies when finding relationships between features. These networks do not necessarily reflect causality, but they are typically concise (with limited indirect effects) and allow for easier identification of the most important relationships. However, with small sample sizes learning Bayesian networks can be difficult. For example, network learning on systems with as few as 20 variables may often be tested using 500 or more samples [11]. Larger and more complex networks may require even more samples for robust inference, which is typically infeasible in complex model systems. Bayesian network inference also does not computationally scale well to large numbers of features [12], though analysis of high-dimensional datasets is at the core of systems-scale, “omics” hypothesis-generating research.

Classifiers, which are algorithms that predict the category of a sample based on data about that sample, are a class of techniques that can perform their task well even with comparatively few samples [13]. This is perhaps unsurprising, since only one feature or value is to be predicted rather than an entire network of connections. This focus only on relationships to one central feature, rather than between all of them, also typically enables classifiers to scale more easily to large numbers of features. However, focusing on just individual relationships to a central feature may ignore information that could provide improved predictions. To this end, Bayesian networks have previously been used to create effective classifiers [14, 15] that exploit this information content. In these Bayesian network based-classifiers, the actual structure of the network is not viewed as important—it is only a means to an end of correct classification – and they thus are typically not assessed.

We hypothesized that if Bayesian network classifiers can be so effective at prediction (even in cross-validation assessment), then they likely contain useful information about the underlying (regulatory) structure in the networks being learned for the classification task, even if that is not an intended focus of the algorithms. The selection of nodes for inclusion in the model and the placement of edges between nodes, while intended merely for classification purposes, may in fact capture some of the most informative underlying structure that we would like to learn for biological interpretation. The fact that there is often some observed phenotype (e.g., a clinical parameter) that one would like to explain based on systems-scale data (e.g., transcriptomics) only further supports the idea of using classifiers as the basis for network learning: the systems-scale data can be used to “classify” the observed phenotype and lead to the learning of a network.

Accordingly, we chose to harness a recently-published tree-like Bayesian network classifier [16] (effective even for small sample sizes) and modify it to learn regulatory networks from biological datasets with comparatively few observations. These constraints are driven by our work in systems biology studies of non-human primate models of malaria, where the number of samples obtained per experiment is typically not greater than 50 but the number of features per experiment is sometimes in the thousands. To our knowledge, the problem of Bayesian structure learning under the constraint of extremely small sample sizes has not previously been considered in depth.

We leveraged the extremely effective predictivity of the previously developed tree-like Bayesian network classifier [16] by refining it to provide less topologically restrictive learning of network structures. While this approach is most applicable for trees with known roots (e.g., networks of genes associated with a specific phenotype as the root), here we show that it can also be applied to networks with an unknown root node (i.e., all nodes are of the same type). We demonstrate the efficacy of this classifier-based structure learning method using simple synthetic models and established reference datasets. We demonstrate that this approach produces reliable and limited predictions of network architecture under constrained sample sizes, with the potential to generate more efficient network models for complex systems. We also apply this methodology to a real complex biological system dataset, analyzing transcriptional data from a non-human primate model of malaria infection to get better insight into the animals’ response to the pathogen challenge.

## Methods

### Background and problem specification

A Bayesian network is defined as B = <S, Θ>, where S and Θ respectively represent a directed acyclic graph and a set of conditional probabilities associated with the graph. Each vertex in the graph is a feature (or a variable), and a directed arc from vertex i to another vertex j shows a direct dependency relationship of feature j on feature i. Feature i is called the parent of feature j, and feature j is called the child of feature i. Specifically, in a Bayesian network feature j is conditionally independent of all vertices that are not its children, given its parents. In this work, we look to learn S from a dataset consisting of M × P measurements (*D*_*real*_, a M × P data matrix), where M is the number of experiments, P is the number of features (or variables), and M < < P.

### Previous tree-like Bayesian Network (BN-TL) classifier

Tree-like Bayesian networks are a subset of Bayesian networks: they meet all of the requirements of being a Bayesian network, but with the additional requirement that all nodes except for one (the root node) have exactly one parent, while the root node has no parents. In recent work, Lin et al. developed a classifier that learned a tree-like Bayesian Network (BN-TL) to perform the classification task [16]. They showed that this method performed as well as or superior to three common Bayesian network classifiers.

Briefly, the BN-TL method constructs a tree by first identifying the feature f* with the most mutual information with the root and places it as a child of the root. It then finds the feature *f’* with the most conditional mutual information with f* given the root and places it as a child of f*. It then places all nodes with conditional mutual information sufficiently close to that between f* and *f’* as children of f*. If there are any features left, a new branch is established in a similar fashion. This process is repeated until all features are added to the tree.

**Fig. 1:**
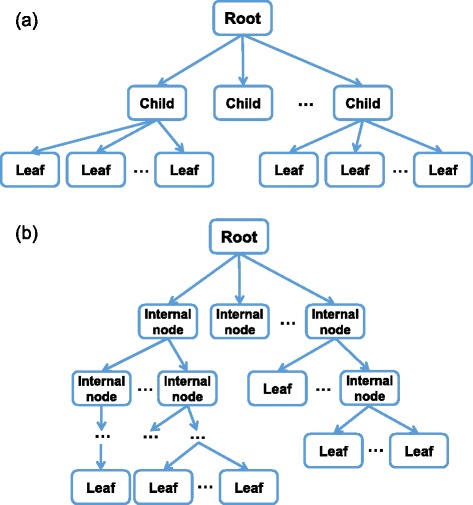
Representation of the topological constraints of two tree-like Bayesian networks. **a** The topology of the previous tree-like Bayesian network classifier (TN-BL) was constrained to three levels: a root, children of the root, and the terminal grandchildren of the root (leaf nodes). Construction of this network did not account for conditional mutual information between siblings. **b** The proposed tree-like Bayesian structure learning algorithm (TL-BSLA) has no constraints on maximum depth of the network and considers the mutual information and conditional mutual information between siblings when creating the network structure

Their method was tested on seven diverse datasets. Its classification performance was shown to be comparable to or better than three common Bayesian classifiers, including naïve Bayes, a general Bayesian classifier learned using a K2 greedy search strategy [17], and another tree-like algorithm [18]. Based on the strength of this approach at predicting classifications, we hypothesized that there is likely significant useful information in this classifier's network, even though that was not the stated goal of the classifier. However, the exact topology of the classifier’s network was not likely to be informative: it was flat, with a maximum of three layers and without consideration of potential relationships between features on the bottom layer (see Fig. 1a). Accordingly, we sought to harness the predictive power of this classifier with more flexible network construction to facilitate learning of generalized tree-like regulatory networks (see Fig. 1b).

### Computational algorithm

Here, we have designed a tree-like Bayesian structure learning algorithm (TL-BSLA) that uses an approach similar to the BN-TL classifier algorithm to infer a generalized tree-like network (Fig. 1b). The goal of this network is to incorporate the most important dependency relationships in the given dataset. The general outline of the algorithm is provided in Table 1.

**Table 1:** Tree-like Bayesian Structure Learning Algorithm (TL-BSLA)

While the most direct application of this approach is to infer trees that explain the behavior of some specified root node (e.g., a phenotype of interest or a known gene of interest), we have also designed the algorithm in a more generalized fashion to allow for the learning of networks in datasets where there is not an obvious root node. If not otherwise provided, the algorithm starts by selecting a reasonable root feature using statistical methods and dataset resampling (using the subroutine **RootSelection**, described in more detail below). After a root is selected, new branches are extended from the root node by adding children to the root. The first candidate child is the node with the largest mutual information (MI) value with the root, where MI is defined as:

1 $$ MI\left(x;y\right)={\displaystyle \sum_{x,y}p\left(x,y\right){ \log}_2\frac{p\left(x,y\right)}{p(x)p(y)}}, $$

From the root, a minimally significant MI value (based on the *ffc_filter* parameter, default value of 0.01) is required to allow the establishment of a new branch, helping to filter out features with minimal relationship with the root node if they exist. For the first established branch, child nodes to be added to that branch are searched for iteratively (**FindLayerNode**), and are then configured into sub-tree structures (**StructureLayerNode**) if they do exist. Once all children to be added to that branch have been identified and appropriately structured, the remaining unassigned node with the largest MI with the root is considered for addition as a new branch; this process is repeated iteratively until all features have been added (or ignored because of their small MI value with the root). The algorithm then returns the learned tree-like structure.

The boldface words above are three functions used repeatedly in the algorithm. Below we provide more details on each function.

- **RootSelection***:* If there is not a specified phenotype to be described with the dataset or an obvious root node for the system, then the first step of the TL-BSLA approach is to select a reasonable root node from the given feature set. As illustrated in Table 2, this procedure consists of four steps:

**Table 2:** RootSelection subroutine of TL-BSLA

1. Create a noisy dataset (D_noisy_, a M×(αP) matrix), where α is the ratio of synthetic noisy features to real features. A synthetic noisy feature is created by randomly permuting the M observations of a real feature; this is done α times for each of the P real features.
2. Treat every real feature as the root node temporarily, and consider two datasets D_real-1_ and D_noisy_, where D_real-1_ is the original real dataset with the observations for the current root node temporarily removed. For each feature in D_real-1_ and D_noisy_, calculate its significance level ***S***_***i***_ with the current root node as the following:
  2 $$ {S}_i=\frac{M{I}^{true}\left({f}_i; current\_ root\right)- mean\left(M{I}^{perm}\left({f}_i; current\_ root\right)\right)}{std\left(M{I}^{perm}\left({f}_i; current\_ root\right)\right)}, $$
  where *MI*^*true*^ represents the MI value between feature *f*_*i*_∈ D_real-1_ or D_noisy_ and the current root, and *MI*^*perm*^ represents the mutual information of randomly permuted *f*_*i*_ and the root. N_P_ permutations are used for each *f*_i_ (e.g. N_P_ = 300), and the mean and standard deviation of the permuted MI are used to calculate *S*_*i*_. This captures the relative significance of the relationship between *f*_i_ and the root given the specific distribution of data in *f*_i_.
3. For each temporarily selected root, compare the *S* distribution for all *f*_*i*_∈ D_real-1_ to the *S* distribution for all *f*_*i*_∈ D_noisy_ (e.g., via a *t*-test). This captures the overall significance of the root’s relationships with all nodes given the specific distribution of data in D_real_.
4. Select the feature with the largest difference between its two *S* distributions (e.g. the one with the smallest *p*-value in a *t*-test) as the final root. This allows for the network that is inferred to represent the strongest overall relationships in the data.

This function returns the selected root and a revised set of nodes D’ which includes all of the original nodes except for the root.

b) **FindLayerNode** Given the nodes ***f***_***bottom***_ (***f***_***bottom***_ may be a single node or a set of multiple nodes) at the bottom layer of the current branch, the first step in this procedure is to determine whether there exist any child nodes that could continue this branch in the next layer. A candidate feature *f*_*i*_ will be considered as a possible child of some node in the current bottom layer if MI’(*f*_*i*_; ***f***_***bottom***_) ≥ *ffc*, where *ffc* is a user-determined parameter, MI’(*f*_*i*_; ***f***_***bottom***_) is the maximum value of MI(*f*_*i*_; ***f***_***bottom***_) and the conditional mutual information (CMI) of *f*_*i*_ and ***f***_***bottom***_ given the parent of ***f***_***bottom***_ (CMI(*f*_*i*_; ***f***_***bottom***_ |***f***_***bottom****_****parent***_)), and MI(*f*_*i*_; ***f***_***bottom***_) is the maximum value of MI(*f*_*i*_; *f*_*bottom,j*_) for all *f*_*bottom,j*_∈ ***f***_***bottom***_.

Instead of MI, the MI’ value is used here because it not only accounts for the direct impact of the parent nodes, but also considers the indirect influence originating from the grandparent nodes. The numerical value of the parameter *ffc* is a user-determined parameter. Here, we use 0.3 based on empirical experience and suggestions from the previously-developed Bayesian network classifier [16]. Although the selected value of *ffc* may affect the ultimate inferred structure, parameter sensitivity analysis (discussed in detail in Results) has shown there to be a fairly broad interval of *ffc* around 0.3 over which the algorithm’s results are insensitive.

c) **StructureLayerNode** The purpose of this procedure is to arrange the candidate child nodes identified in **FindLayerNode** into a sub-tree structure using the nodes currently in the bottom layer of the current branch as potential parent nodes. As schematically described in Table 3, the input of this procedure includes the current bottom nodes (***bottomNodes***), which are considered as the temporary roots for the sub-tree, the corresponding parent nodes of the bottom nodes (***parentsOfBottom***), and the candidate pool for searched child nodes (***child_pool***). The configuration starts with the calculation of MI’(*bottomNodes*_*j*_; *f*_*i*_) for all *bottomNodes*_*i*_∈ ***bottomNodes*** and *f*_*i*_∈ ***child_pool***. For each *f*_*i*_, the *bottomNodes*_*j*_ with the largest MI’ value with *f*_*i*_ is identified as a potential parent of *f*_*i*_; we refer to these pairs as (*bottomNodes*_j_*, *f*_i_*). Then, each *bottomNodes*_j_* is connected by an arc to the *f*_i_* with the greatest MI’ value with *bottomNodes*_j_* from among all of its potential children *f*_i_*. For *bottomNodes*_j_^*^ with multiple potential children, an additional independence test is used to determine if additional children add sufficient independent information to allow their inclusion in this layer: if CMI(*f*_*k*_; *f*_i_* | *bottomNodes*_j_^*^) ≤ *ffc_independent* (default value of 0.1), an arc is created between *bottomNodes*_j_^*^ and *f*_*k*_. That is, *f*_*k*_ is considered to be another child node of *bottomNodes*_j_^*^ because it is (sufficiently close to) conditionally independent of the other children in the layer and thus should not be a child of those nodes. This process is continued iteratively until all nodes returned by **FindLayerNode** have been added to the tree.

**Table 3:** StructureLayerNode subroutine of TL-BSLA

### Literature and synthetic datasets

Four examples were used to evaluate the performance of the proposed TL-BSLA. We developed a simple synthetic network (17 nodes, 15 edges) with a true tree structure except for a single node that is not connected to the rest of the graph. In this work we refer to this network as synthetic-tree; the true network is illustrated in Additional file 1: Figure S1. The other three example networks used in this work are published networks widely used in structure learning literature: the Child system, the Alarm system, and the Asia system (http://www.bnlearn.com/bnrepository/). The Child system is a tree-like network with 20 nodes and 24 edges, but is not exactly a tree. The Asia and Alarm networks are less tree-like networks (8 nodes, 8 edges and 37 nodes, 46 edges, respectively) used to assess the algorithm’s performance on data drawn from underlying networks more similar to real “omics” data. Data was generated based on the probabilities defined by each model network, with all variables being discrete.

### Experimental data

Transcriptional data was used from a recent malaria challenge experiment in five rhesus macaques (*Macaca mulatta*).

### Ethics statement

The experimental design of this experiment involving rhesus macaques (*Macaca mulatta*) was approved by the Emory University Institutional Animal Care and Use Committee (IACUC) under protocol #YER-2001892-090415GA.

### Malaria challenge experimental methods

The experimental protocol was similar to that used in our previous malaria challenge experiment [19], with four noteworthy exceptions: there was a longer follow-up period for measurements, complete blood count (CBC) profiles were determined every day, there was no biotinylation of erythrocytes, and *Plasmodium cynomolgi* sporozoites were used for the experimental infection. Bone marrow aspirates were taken under anesthesia with ketamine at seven time points over the course of approximately 100 days, corresponding to baseline, peak of parasitemia, treatment of blood-stage parasites, and during and after relapse. Transcriptional profiles were obtained by sequencing on an Illumina HiSeq2000 at the Yerkes National Primate Research Center Genomics Core. Additional details on the infection protocol, sampling protocol, and methods for initial processing of transcriptional data are available in Additional file 1: Supplemental Methods.

### Experimental data processing

Since the transcriptional profiles consisted of continuous variables, they were first discretized. This is a common data processing step, as it decreases the computational complexity and the minimum number of samples required for accurate structure learning. We have previously described methods for discretization of continuous data and their potential impact on learned networks during structure learning [8, 20]. Here, we have taken a simplified approach for our proof-of-principle analysis of a malaria-based dataset, using an equal-quantile discretization to evenly divide the values for each variable into high, medium, and low values. Genes describing the recently identified axes of variation across large-scale human population human cohorts were used as a starting point for analysis, to facilitate data interpretation and network construction [21].

### Comparator algorithm

Our main goal in this work was to test the hypothesis that the information contained in a Bayesian network classifier would be sufficient to provide informative learning of the actual underlying Bayesian network. To provide a benchmark for acceptable performance in network learning, we selected the Sparse Candidate Algorithm (SCA) [22] as implemented in the Causal Explorer package [23]. Numerous algorithms have been published for structure learning of Bayesian networks, with no conclusively optimal algorithm. We selected SCA as the main comparator because it is widely-used and generally performs well, it is effective at handling reasonably large-scale networks (critical for systems biology datasets), and it typically provides better positive predictive value in its inferred networks (fewer false positives per predicted positive). The avoidance of false positives is particularly important for the design of validation experiments in complex model systems. In previous work [24] we have found that many other algorithms (for example, PC [25], Max-Min Hill Climbing [11], and Three Phase Dependency Analysis [26]) often learn many more false positives than true positives when sample sizes are limited. SCA learns a significant fraction of those true positives with many fewer false positives, making it a desirable choice.

## Results

### A classifier-inspired algorithm can effectively learn tree-like network structures

As described in greater detail in the Methods, we have developed a tree-like Bayesian Structure Learning Algorithm (TL-BSLA) by building off the success of a previously published tree-like Bayesian network classifier [16]. We removed some topological limitations from the existing Bayesian network based-classifier and used conditional mutual information to appropriately arrange the nodes in the network.

Four example networks were used to evaluate the performance of the proposed TL-BSLA relative to a benchmark algorithm. The networks included a simple synthetic-tree network and three widely used literature networks with tree-like (the Child system) and non-tree-like (the Alarm and Asia systems) structures. For each example, 10 randomly generated datasets were tested to reduce the impact of dataset-specific biases introduced by sampling; each dataset was analyzed using our proposed TL-BSLA and the well-known Sparse Candidate Algorithm (SCA) structure learning method [22] for sample sizes ranging from 50 to 500 observations. More detailed justification for using SCA is provided in the Methods, but its key feature is that it typically provides good positive predictive value (fewer false positives per predicted positive).

**Fig. 2:**
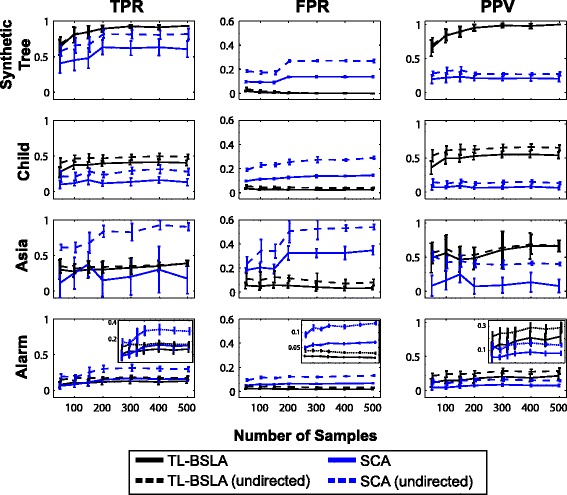
TL-BSLA performs consistently better than SCA in four example systems. The true positive rate (TPR), false positive rate (FPR), and positive predictive value (PPV) are shown for four representative networks. Black lines show performance of TL-BSLA, blue lines show performance of SCA. Dashed lines represent calculations without considering the direction of connections when assessing their correctness. TL-BSLA is almost universally better than SCA, with the exception of TPR for the Asia and Alarm networks where the directionality is not accounted for in assessing correctness. In these cases, the much higher FPR of SCA outweighs its potentially better coverage of true positives, as evidenced in the superior PPV curves for TL-BSLA. For PPV, all performance metrics across all networks (directed and undirected) are statistically significant (*p* < 0.05, two-tailed *t*-test) except for the 50 and 150 sample sizes for the Asia network for the undirected case. Error bars are one standard deviation

Three metrics were used to assess the accuracy of learned structures for each algorithm: (1) true positive rate (TPR), the fraction of all actual edges that are correctly predicted by an algorithm; (2) false positive rate (FPR), the fraction of all actual non-edges that are incorrectly predicted by an algorithm to be edges; and (3) positive predictive value (PPV), the fraction of predicted edges that are actually edges. Worth noting is that PPV is the most relevant metric for the purposes of model validation, as it determines the likelihood of success of often extremely expensive or difficult validation experiments: a low PPV will confound the experimental validation of network structure and have significant costs. For identification of true positives, we considered two cases in all analyses: if a learned edge needed to be the correct direction in order to count as a true positive, or if the correctness of each edge was determined without consideration of directionality. The same root was used for all analyses of a given network in order to provide sufficient consistency for comparison; roots were selected as described in the Methods. The results of this evaluation are presented in Fig. 2.

In the synthetic-tree system, TL-BSLA correctly recovered almost all of the correct connections even when the sample size was rather small (e.g., the average TPR was 80 % when the sample size was 100; Fig. 2). In comparison, the SCA approach achieved a lower TPR than TL-BSLA, regardless of whether directionality was considered in assessing the accuracy of the networks. When directionality was considered, SCA performed much more poorly than TL-BSLA. This was particularly noticeable at low sample sizes: SCA recovered no more than 50 % of the true edges when the sample size was below 200. Even without considering directionality, the performance of SCA on this simple system was still significantly worse than that of TL-BSLA. Moreover, the average FPR for SCA was always greater than 10 % (for directed edges) and 20 % (ignoring directionality), which was at least 4-fold higher (and often an order of magnitude higher) than that of TL-BSLA. Accordingly, the PPV for TL-BSLA was much better for the synthetic-tree network.

The TPR for TL-BSLA was consistently higher than for SCA for the Child system whether or not the directionality of the learned edges was considered (Fig. 2). The magnitude of the difference was also fairly consistent across the range of sample sizes; most importantly, there are significant differences between the two methods at low (50 or 100) sample sizes. The TL-BSLA also had a much lower FPR than SCA, indicating that fewer incorrect edges were learned by the algorithm. As a result, the PPV of the TL-BSLA was again significantly better than that of SCA.

We also analyzed whether the networks inferred were sensitive to changes in the input datasets. It has previously been observed in biomarker discovery [27] and in network inference [8] that resampling of data can yield different outputs for machine learning and network inference algorithms. To assess this we followed a previously published approach to assess robustness to resampling [27]. From a fixed set of 500 samples for the Child network, we selected subsets of 125 samples; we used TL-BSLA to learn networks for 100 such resampled sets, with each set having no more than 30 % similarity to any of the others. The average number of connections found per dataset was 19. Using the TL-BSLA, 18 connections were found in at least 60 % of the resampled datasets, suggestive of robust structure learning by TL-BSLA. 13 of those connections were true positives (11 with correct directionality). These results are consistent with the TPR and PPV performance shown in Fig. 2. On the other hand, only 4 connections were found in every subsample, and 25 connections were found in at least one and at most 10 % of subsamples (22 of those 25 were false positives). Thus, while there is variability in the networks found based on the input dataset just from resampling, TL-BSLA is capable of consistently picking out a core of true positive connections. This robustness may decrease, though, if real biological samples have significantly greater noise than the synthetic noisy data used here. Additionally, we note that in the case where many samples are available, a resampling technique such as this can be useful to identify the highest-confidence and most robust connections for further experimental and computational investigation [8, 27].

### A tree-like algorithm can also perform well on non-tree-like underlying structures

As for systems whose underlying structures do not resemble trees, such as the Alarm and Asia systems, Fig. 2 shows that the TL-BSLA performed competitively with, and in some important respects better than, SCA. In both datasets, for the identification of edges with the correct directionality, the true positive rates for the two algorithms were statistically indistinguishable. In both cases the TL-BSLA was more robust to the reduction of sample size to small values (or conversely, that the performance of SCA was likely to improve faster than that of the TL-BSLA as sample size increased to levels typically beyond that available for complex model systems). When edge directionality was ignored, the performance of SCA improved much more than that of TL-BSLA, and was statistically significantly better for all sample sizes in the Asia system. However, we note that at small sample sizes, the true positive rate ignoring directionality was statistically indistinguishable for the larger, more realistic Alarm system.

Importantly, though, the false positive rate for the TL-BSLA was much lower (two to four-fold lower, across all sample sizes and regardless of directionality consideration) than that of SCA. This ultimately resulted in PPV performance such that TL-BSLA was significantly better at learning connections than SCA across sample sizes, with the difference even more prominent when the directionality of edges was considered. Taken together, this suggests that the use of a classifier-based Bayesian network learning strategy that is computationally efficient may be a viable replacement for existing network learning algorithms.

Based on the across-the-board improved PPV performance of the TL-BSLA and the details of how it works, it is worth noting that the main benefit of SCA (its ability to capture a greater fraction of the true positive edges) can likely be captured through iterative application of TL-BSLA. Once a root is set for TL-BSLA, areas of the network that are essentially insulated from that root and its subnetwork (or are otherwise independent of that root) will not be considered. This is appropriate for classifier-based tasks, but for the purposes of learning a complete network from large-scale data suggests that by initiating the algorithm with a separate root that was not used in the initial inference, additional true positive edges are likely to be discovered (with likely similar PPV), resulting in even further improved performance of the TL-BSLA.

### Network learning performance is not sensitive to the choice of ffc

**Fig. 3:**
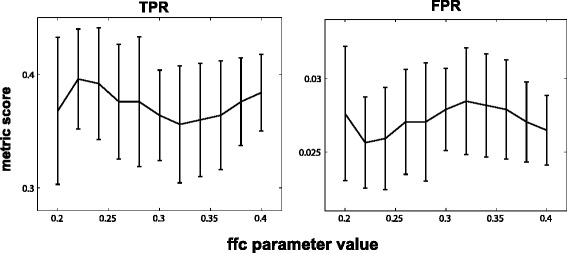
Sensitivity analysis of *ffc* shows less significant impact than random variability. The Child network was analyzed with 100 samples, 10 times each for *ffc* parameter values ranging from 0.2 to 0.4. The variability induced by changing *ffc* (range of TPR and FPR across all parameter values) is smaller than the variability from different random datasets being used for structure learning (error bars for any given *ffc* value). This suggests that there is a broad optimum of *ffc* values and that the value used in this work is a reasonable one (and perhaps not even optimal). TPR: true positive rate; FPR: false positive rate. Error bars represent one standard deviation

We found that the parameter that most directly affected structure learning results was *ffc*, used to determine which nodes are children of the current bottom layer nodes. It is a user-defined parameter with an optimal value that is possibly data-specific. In our simulations, we used *ffc* = 0.3 based on our initial explorations and some empirical experience from previously published work [16]. However, it is important to assess the sensitivity of algorithm performance to critical parameters so that over-fitting and inappropriate comparisons are avoided. We assessed the accuracy of TL-BSLA when varying *ffc* values over a factor of 2 (from 0.2 to 0.4, see Fig. 3). For example, for the Child system with 100 samples, we found that the variation induced by different *ffc* values (the maximum difference for average TPR induced by different *ffc* is less than 10 %) was smaller than the variation induced by different datasets (e.g. the TPR across 10 datasets can vary by over 20 %). This *ffc*-induced variation became even smaller as the sample size increased (Additional file 1: Figure S2). Under the constraint of small sample sizes, the variation induced by even substantial changes of *ffc* was thus not substantial. In fact, 0.3 was not even necessarily the optimum value of *ffc* (see Fig. 3), but we used it successfully for four diverse datasets (in terms of both size and topology) being studied here. We also performed a similar parameter sensitivity analysis for SCA to confirm that the results in Fig. 2 were not due to poorly chosen defaults. This analysis is presented in Additional file 1: Figure S3, showing that the performance of SCA was not highly sensitive to its parameters and that changing parameter selections did not allow SCA to perform as well on the Child system as the TL-BSLA.

### Generalization of the strategy by selecting a root

While one of the primary goals of our algorithm is to generate networks that explain some single important phenotype from a dataset (and thus used as the root node), in some cases there may not be an obvious choice for that root node. Existing training sets for classification problems typically do not include associated regulatory networks, and thus there is no way to assess the accuracy of networks we would predict for those training sets. Instead, we needed to use training sets designed for assessing network learning, which meant that there would not necessarily be obvious roots for the networks. Accordingly, we devised a strategy to identify a reasonable root node based on a statistical treatment of the specific dataset being analyzed. Our root selection procedure, **RootSelection** (see detailed descriptions in Methods), resamples from the existing dataset and uses dataset permutations to identify a reasonable, statistically meaningful root for learning a tree-like Bayesian network. The root is identified as the node that has the most significant mutual information with the true features relative to a set of randomly permuted features.

**Fig. 4:**
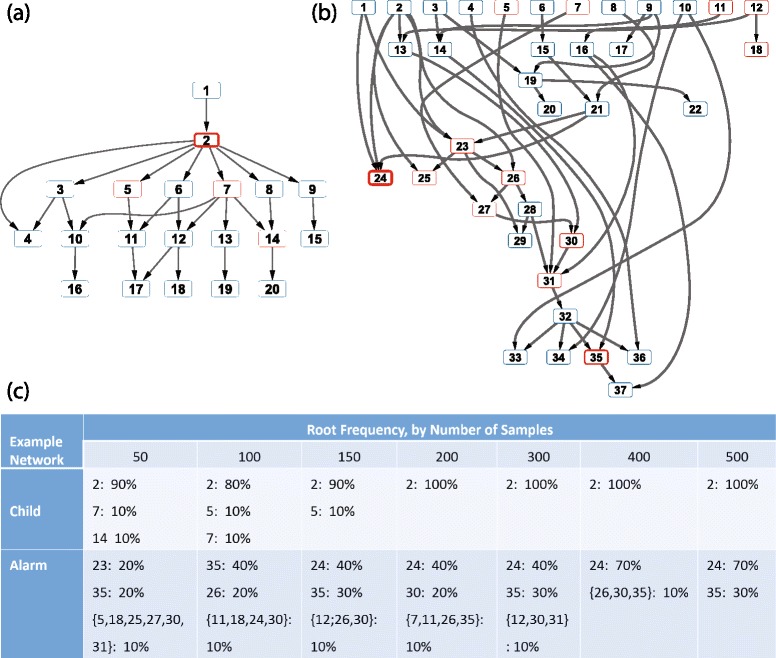
The tree-like Bayesian Structure Learning Algorithm can select a root for structure learning in tree-like or non-tree-like networks. Roots were selected automatically for two representative networks across a range of sample size limitations: **a** the tree-like Child network and **b** the non-tree-like Alarm network. Any node ever selected as a root has a red outline, where increasing line width indicates increasing frequency of selection as a root. Nodes never selected as a root have blue outlines of fixed width. **c** A quantitative summary of the root nodes selected, as a function of sample size. Selection from a tree-like structure is straightforward and consistent; from a non-tree-like structure there is increased variability, but reasonable roots (excluding directionality) are typically chosen. Feature 24 was used as the root for previous Alarm network learning work. It is worth noting that selection of a better root could improve the TL-BSLA’s TPR and PPV even further

We used the Child and Alarm networks (as representatives of tree-like and non-tree-like underlying networks) to assess the performance of our dataset-specific, unbiased root selection approach. For each example, we considered the impact of varying the number of observations (samples) for the features from 50 (a reasonable value for many “omics” approaches) to 500. For each number of observations, we used 10 different randomly generated training datasets. The selected roots for each example are summarized in Fig. 4.

The root selection approach performed quite robustly in the tree-like networks. For the Child system (as shown in Fig. 4a), node 2 was consistently returned as the root for all sample sizes. Even for small sample sizes (e.g., 50), node 2 was selected as the root most of time. While node 1 is actually the real root, node 2 is obviously a reasonable second option for root selection based on the topology of the network. There was little sensitivity of selected root to sample size, which is particularly valuable for applications to small sample datasets.

For the Alarm system, there was not such a strong consistency of root selection, though the results were still fairly insensitive to sample size. For different training datasets with the same number of samples, different root nodes were often selected; as shown in Fig. 4b, the roots selected most often across all sample sizes were nodes 24, 35, 30 and 26. Only for a sample size of 50 was the selection of root nodes particularly variable. Since the network topology of the Alarm system is not tree-like, there is no single “correct” root that characterizes the entire network, and each of these seems on initial inspection to be a potentially reasonable selection, especially if directionality of edges is ignored. To recover the structure of the whole system, multiple trees with different roots could be combined together in some form (as discussed above). While that is an interesting future direction, here we focus on finding a single strong sub-network with constrained sample size to demonstrate the potential for using classifier-based algorithms to learn network structure.

### Data reduction for omics-scale datasets

For high-throughput and “omics”-scale studies, such as transcriptomics and metabolomics, datasets typically contain relatively few samples but thousands of features. In general, this can make it harder to identify the (likely few) features that are relevant to determining the phenotype or structure of the system because the desired signal may be buried in multivariate noise. For small sample sizes, a further problem is that supervised identification of the important features can often lead to overfitting, where some linear combination of features can explain even random results or classifications. Moreover, increasing the number of features also increases the computational cost for essentially all types of data analysis. In order to make our structure learning method useful for such datasets, we developed an additional screening step (consistent with the approaches used within the TL-BSLA) to exclude features likely irrelevant to the selected root. This method was not used in the above assessments, and is considered as a useful addition to the algorithm proposed in Table 1.

Specifically, for each feature in the dataset (*f*_i_), it is included in the network if it satisfies *S*_i_ ≥ *threshold*, where *S*_i_ is as used in the **RootSelection** subroutine and is defined in Equation (2). *S*_i_ represents the significance of the mutual information between *f*_i_ and the root, given the specific distribution of data in *f*_i_. In this work we used a *threshold* value of 2.6, based on statistical arguments, previous literature [28], and empirical exploration. The *S* value is essentially a z-score on the mutual information of a feature with the root based on a background of permuted data for that feature; thus, a *threshold* of 2.6 on a zero-mean, unit-variance normal distribution corresponds to a one-tailed significance of 0.005, where a significance lower than the typical 0.05 threshold was selected to limit false positives due to multiple hypothesis testing. Changes in *threshold* change how conservative the feature inclusion is, and thus affect the true positive and false negative rates; variation of *threshold* was not explored due to its statistical interpretation.

We tested the performance of this screening step by adding 10-fold manually-created noisy features to the set of real features in the example datasets. These noisy features were generated via permutation of the observations within each real feature. Using the Child system as a representative example (see summarized results in Table 4), with node 2 as the root, we found that over 50 % of the real features were included as significant features when the sample size was 50. In contrast, only 2.5 % of the noisy features were selected as significant. As the sample size increased, the percentage of real features selected for inclusion gradually increased to over 80 %, indicating that most of the real (or relevant features) had been correctly selected. Interestingly, the percentage of noisy features remained at approximately 3 % even with an order of magnitude more samples. This again supports the idea that our overall structure learning approach is effective for the case of complex model systems with limited numbers of samples. Results for the synthetic-tree and Alarm systems (see summarized results in Additional file 1: Table S1 and Additional file 1: Table S2) were similar to those of the Child system, indicating that our proposed screening step can generally exclude noisy features that are irrelevant to the root across different types of underlying network structures. Thus, once the root is determined (whether through *a priori* knowledge or a statistical approach as described above), we can focus on the features most relevant to the root with a concomitant reduction in computational complexity.

**Table 4:** Features in the child network selected for model inclusion using a dimensional-reduction screening procedure, with node 2 (selected automatically) as the root

Sample size	Indices of features identified as significant	Fraction of real features selected	Fraction of noisy features selected
50	Real features: 3,4,5,6,7,8,9,12,14,15,20	58 %	2.5 %
	Noisy features: 96,113,119,174,175		
100	Real features: 1,4,5,6,7,8,9,11,12,14,15,20	63 %	0.5 %
	Noisy features: 161		
150	Real features: 3,4,5,6,7,8,9,11,12,14,15,20	63 %	2 %
	Noisy features: 70,79,116,208		
200	Real features: 3,4,5,6,7,8,9,11,12,14,15,20	63 %	1 %
	Noisy features: 75,94		
300	Real features: 3,4,5,6,7,8,9,10,11,12,14,15,20	68 %	3.5 %
	Noisy features: 121,126,198,205,207,209,211		
400	Real features: 1,3,4,5,6,7,8,9,10,11,12,14,15,20	74 %	2.5 %
	Noisy features: 31,55,94,113,195		
500	Real features: 1,3,4,5,6,7,8,9,10,11,12,13,14,15,19,20	84 %	3.5 %
	Noisy features: 22,122,152,157,166,192,218		

### Application of TL-BSLA to analyze transcriptomic data

To apply this approach to the analysis of real systems biology data, we used results from a recently-completed experimental challenge of five rhesus monkeys (*Macaca mulatta*) with the malaria parasite *Plasmodium cynomolgi*. Transcriptional profiles were measured from bone marrow aspirate samples that were taken seven times over the course of three months after infection. We used these transcriptional profiles as the basis for the construction of a Bayesian network. We used the recently described axes of common variation across large-scale population human cohorts [21] to provide a more focused analysis on transcripts likely to be informative in describing the animals’ response.

**Fig. 5:**
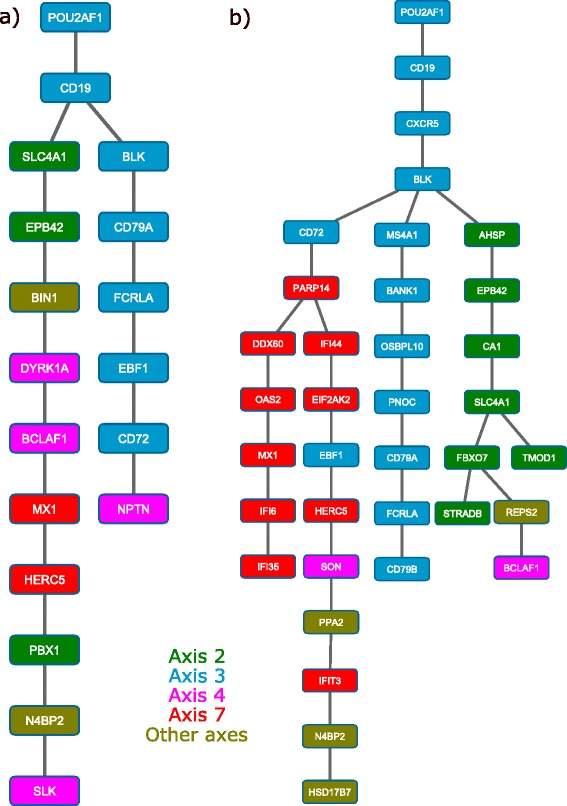
Tree-like Bayesian networks learned from transcriptional data of a malaria challenge experiment in *Macaca mulatta*. Networks were learned using blood informative transcripts [21] to focus on potential Axes of variation in the transcriptional data. **a** Using the ten blood informative transcripts as originally published, two branches emerge that best describe the root (selected automatically and which is from Axis 3), consisting of other genes from Axis 3 and a combination of multiple genes from Axes 2, 4, and 7. **b** Using the top 25 genes from each Axis to build a network based on the same root, the relationship between the Axes becomes even more evident, as both Axis 2 and Axis 7 contribute the dominant genes in parallel branches of the tree, suggesting significant but distinct mutual information with their parent and ultimately with the root. These relationships were not evident using standard multivariate and clustering analyses, and were not expected *a priori* based on previous descriptions of the axes of variation and the fact that the gene lists were derived from whole blood, not bone marrow aspirate, transcriptional profiling analyses

Figure 5a shows a representative inferred network from the data, demonstrating the flexible nature of the networks that can be inferred using TL-BSLA: dependency relationships several levels deep are identified. We began with a simplified analysis using previously defined “blood informative transcripts” from previous work as best describing uncorrelated Axes of variation in whole blood transcriptional profiling of healthy subjects [21]. There are seven main Axes most strongly observed in both human and macaque samples (based on previous work; Axes 8 and 9 are weaker and are typically only observed in much larger datasets); we compiled together the ten blood informative transcripts for each of these Axes for input to the TL-BSLA. The genes in the network were selected using the dimensional reduction scheme described above, yielding a network of manageable size for visual and biological interpretation. The root was automatically selected from the data, using the approach described in the Methods. There were two main branches in the tree: one branch almost exclusively consisting of Axis 3 transcripts, and one that is a combination of multiple transcripts from Axes 2, 4, and 7. While this network indicated potentially interesting relationships between the Axes, it also suggested that deeper exploration by including more genes from each Axis would help to better distinguish potential relationships from noise. We thus rebuilt the network from the same root instead using the top 25 genes from each Axis. This deeper analysis of the Axes made the relationships within the tree even more evident (Fig. 5b): Axes 2 and 7 have a significant interaction with Axis 3, which is the root of the tree. Each of these three Axes has a branch almost exclusively consisting of only members of that Axis, suggesting a coherent, significant relationship with the level of the root gene.

## Discussion

Network structure learning is a useful tool to help identify unknown regulatory or causal relationships between variables (features) in a system. With the rise of systems-scale “omics” data over the past two decades, structure learning methods have been applied with increasing frequency to biological systems for the discovery of new modules, pathways, and regulation. However, in many cases the number of samples available in “omics”-scale studies is small, particularly in the case of complex model systems (such as non-human primates). In addition, while these techniques often include measurements for many variables or features (genes, metabolites, etc.), often only a small fraction of them are directly relevant to the phenomenon being studied. Secondary or indirect effects may make many more genes appear to be highly correlated with each other and with a phenomenon of interest, and these indirect effects hinder the identification of the real regulatory relationships in complex systems.

### A tree-like network inference approach based on classifier learning algorithms

To address the issues associated with network learning in complex model systems, we hypothesized that Bayesian network-based classifiers that have been proven to be effective with few samples and with many features may, within their networks, have the potential to capture important regulatory structure in addition to their classification prediction. Accordingly, we created a new structure learning method, the tree-like Bayesian structure learning algorithm (TL-BSLA), which refined a previously demonstrated effective tree-like Bayesian network classifier by removing limitations on network topology (though still within the constraints of a tree-like network). We chose to focus on Bayesian network structures because they typically provide a more succinct representation of regulatory interactions than correlation-based networks, and because the relationships between features are highly suggestive of direct interaction or regulation, each of which are valuable properties for driving validation experiments or mathematical modeling efforts.

### Iterative structure arrangement steps enable learning of network connections

In the TL-BSLA, we improved upon the previous classifier-based approach in a number of ways. We refined the arrangement of nodes within branches to more accurately reflect the relationships between those nodes as opposed to just their (conditional) mutual information with the root node. This entailed developing a strategy to iteratively select nodes for inclusion in a branch and to arrange their topology in a manner reflective of likely interactions based on mutual information and conditional mutual information.

Using four different networks as examples, we supported our hypothesis on the utility of network-based classifiers for learning regulatory structure via the effectiveness of the proposed TL-BSLA to infer reasonable regulatory networks for a variety of different underlying topologies. For the systems with tree-like structures (e.g., synthetic-tree and Child), even with a limited number of samples (50 or 100 samples), the algorithm could recover most of the correct connections with a lower false positive rate than a commonly used structure learning algorithm, SCA.

While it may not be surprising that an algorithm designed to learn trees can perform fairly well for underlying networks that do in fact resemble trees, we posit that it is still surprising that it performs substantially better than an existing, widely-used structure learning approach like SCA. The underlying networks are sparse and are ultimately rather simplified Bayesian networks; one would expect such networks to actually be fairly easy to infer for an algorithm like SCA. It is certainly possible that the constraints of being a tree-like structure contribute to the ability of TL-BSLA to infer accurate networks with fewer samples. Nonetheless, given that TL-BSLA can find more true positives with fewer false positives without any external information about the true root of the system, this suggests its potential wider utility, particularly for fairly simple networks.

In networks without a tree-like structure (i.e. Alarm and Asia), the algorithm was still able to recover a substantial portion of the original network. More importantly, we found that the false positive rate of TL-BSLA was much lower than SCA, which itself typically has a low false positive rate [24]. This yielded a better positive predictive value for TL-BSLA, which makes it a more effective strategy for learning networks of relationships in biological datasets under the constraint of small sample size.

### Addition of other functionalities enables broader applicability of the algorithm

We addressed the inclusion of nodes via a feature selection step in the algorithm. The previous classifier excluded features based on a user-defined parameter that eliminated a fixed fraction of the features based on mutual information with the root. While this certainly makes sense for learning a classifier (only those features most directly related to the root node should be included), having a user parameter that so significantly affects the members and structure of a network is undesirable for network learning. We used statistical approaches to identify the features with statistically significant mutual information with the root, which retains most of the relevant features with fairly low inclusion of irrelevant features. As shown in Table 4, this screening step shows good performance in separating relevant and non-relevant (noisy) features.

We also addressed the issue of root selection to attempt to generalize the algorithm to network learning without a target phenotype to be predicted. We used mutual information-based statistical approaches to identify the best candidates for roots, with robust selection in the case of underlying tree-like structures and reasonable selection (though variable with different datasets) for underlying structures that are not tree-like.

### Application to a malaria transcriptomics dataset provides leads on biological insight

We then applied this approach to a transcriptional dataset of bone marrow aspirates from a group of five *M. mulatta* infected with the simian malaria parasite *P. cynomolgi*. Focusing on genes representing common Axes of transcriptional variation, we applied all aspects of our network inference approach: selection of significant features based on mutual information relative to resampled and permuted data, identification of a root node based on the significance of the mutual information between the root and the rest of the features, and then learning of tree-like network structure. The algorithm automatically constructed a network that was deeper than it was wide (suggesting somewhat pathway-like behavior), although multiple independent branches within the network were learned.

An initial network defined by the top ten most informative transcripts from each Axis suggested a possible relationship between four of the Axes; including more genes in the analysis, it was clear that three of the Axes (2, 3, and 7) had a significant relationship. Each of them dominated a different branch in the network, showing that they had significant relationships to the root gene (which is in Axis 3), but that the relationships for each Axis to the root gene were different (since their separation into different branches was based on unique conditional mutual information).

The Axes of variation represent sets of genes that are positively co-regulated in peripheral blood data sets in humans, where each Axis tends to capture an aspect of blood and immune system biology. Notably here, Axis 3 is enriched for B-cell signaling, Axis 2 for erythropoiesis, and Axis 7 for interferon signaling. These Axes are not strongly evident in the bone marrow since the major blood cell types have not yet differentiated, but the tree-like Bayesian network nevertheless recovers nascent relationships. It is particularly notable that Axis 3 falls out as a separate branch, since there is no sign in these graphs of Axis 1, which largely captures T-cell signaling, nor of Axis 5, which is closely related to neutrophil activity and inflammation. We would thus argue that our algorithm is capable of capturing the earliest stages of cellular differentiation during leukopoiesis when seeded with genes that are markers for the mature cell types.

This finding is particularly noteworthy for a number of reasons. First, the Axes of variation as originally derived had fairly low covariation with each other, with a few exceptions. However, none of those exceptions were observed here, and relationships between Axes 2, 3, and 7 were not previously observed. Second, the Axes were derived from whole blood transcriptional profiling, so there was not an expectation that the same variation should be seen in bone marrow aspirate transcriptional profiling. Their observability in bone marrow aspirates supports broader utility of this approach to transcriptional analysis. Finally, and most importantly, the relationship structure between Axes 2, 3, and 7 was not identified from standard clustering and statistical analysis of the transcriptional data (analyses not shown). Based on standard multivariate analyses there was not an obvious relationship between these Axes; only through the consideration of conditional mutual information and a network of interactions between genes were we able to identify robust relationships between Axes. Thus, the Bayesian, tree-like network analysis contributed uniquely to understanding and interpretation of the data.

Thus, by identifying the likely expression relationships in our experiments of these genes revolving around related themes (B-cell signaling, erythropoiesis, and interferon-mediated response), the network-based analysis has contributed to interpretation of the data and ultimately to directing future efforts in our studies of the host-pathogen interaction in malaria using non-human primate models.

### Limitations and caveats

The requirement for our learned structure to be tree-like is an inherent limitation to our approach, as biological networks are not necessarily tree-like. There could be significant cross-talk or combinatorial regulation on a given node in a true biological network. However, the networks learned by TL-BSLA are a reasonable approximation even to underlying networks that are not strongly tree-like. Moreover, multiple trees inferred starting from different roots could potentially be pieced together to provide a more complex network representative of multiple subnetworks but that is not tree-like. (This would also mitigate the lower true positive rate of TL-BSLA in non-tree-like networks, with its higher positive predictive value supporting the potential of this approach.) If nothing else, the tree-like network approach would serve as an excellent starting point for a search-and-score heuristic structure learning algorithm and would help to identify which subset of nodes should be included in such a search.

For our comparator algorithm we used SCA, chosen since it is a well-known and widely-used structural learning algorithm with better avoidance of false positives than many other Bayesian structure learning algorithms (an important aspect of our structure learning goals). Countless other algorithms could have been used as comparators; nonetheless, the commonality to many of those algorithms is their inability to robustly learn networks under the constraint of small sample size. In this sense, SCA is a reasonable representative of existing algorithms, and TL-BSLA stands on its own as learning networks effectively under this constraint. Moreover, an important goal of our work was to validate the hypothesis that methods developed for classifier learning could have significant potential for learning network structure, which we have demonstrated here even if there are other Bayesian learning algorithms that perform slightly better than SCA.

The idea that classifier learning could have significant potential for identifying network structure has been hinted at previously; in fact, one of the algorithms that the previous BN-TL classifier compared itself to explicitly notes the potential for identifying valid relationships between features (in their case, specifically for mass spectrometry data) [18]. However, this algorithm also restricted itself to a very flat topology making it difficult to find deeper, more complex regulatory relationships as is enabled by the TL-BSLA.

We also note that our approach did not exploit the temporal aspect of the samples in constructing the network. This information could potentially enable improved structure learning, whether by exploiting the relationship of consecutive samples or by enabling connections between variables that represent regulatory loops as is possible using dynamic Bayesian networks [29–31]. However, robustly learning dynamic Bayesian networks requires even more samples than learning general Bayesian networks, which is counter to the goal of the TL-BSLA.

Finally, we note that for the transcriptional data, since feature selection and root selection are based on permutations and resampling, replicate runs can yield slightly different results. Multiple runs were performed for the networks in Fig. 5, with the ones presented being highly representative of all of the runs; differences between runs were in the inclusion of a few different genes and resulting slight changes in topology at the bottom of a tree (though the topology at the top of a tree is highly conserved).

## Conclusions

Taking together the novel aspects of our tree-like structure learning algorithm with the validation on transcriptional data from a malaria challenge experiment in a non-human primate macaque model system, we have shown that Bayesian network-based classifiers can be the basis for meaningful inference of regulatory network structure. The algorithm we designed for this task, TL-BSLA, is an effective and useful algorithm for structure learning in systems biology data under the constraint of small sample size and is better than an existing, widely-used structural inference algorithm. We have demonstrated its efficacy for systems exactly meeting its tree-like assumptions, for systems that only slightly deviate from tree-like assumptions, and for systems that deviate substantially from tree-like assumptions. By including data-specific assessment of the significance of mutual information, we have enabled the identification of a reasonable root for an arbitrary dataset, as well as the identification and elimination of spurious features. We believe this approach has particularly significant promise for the integration of different types of datasets, where some molecular-level explanation (e.g., gene expression) is desired that explains some observed phenotype (e.g., clinical parameter) that can serve as the root of the tree-like structure. This represents a promising addition to the set of tools for probabilistic graphical model and Bayesian structure learning, filling a need for high-confidence analysis of complex systems with few samples and many variables.

### Availability and requirements

**Project name:** Tree-like Bayesian Structure Learning Algorithm

**Project home page:**http://styczynski.chbe.gatech.edu/TL-BSLA (also available as supplementary information for this paper)

**Operation system:** Platform independent

**Programming language:** MATLAB

**Other requirements:** developed on MATLAB R2011a; backwards compatibility unknown

**License:** FreeBSD

**Restrictions for non-academic use:** None.

## Additional file

Additional file 1:
**Supplementary Figures, Tables, and Methods.** (DOCX 993 kb)

## Abbreviations

BN-TL: Tree-like Bayesian network classifier
FPR: False positive rate
PPV: Positive predictive value
SCA: Sparse candidate algorithm
TL-BSLA: Tree-like Bayesian structure learning algorithm
TPR: True positive rate

## References

[1] Barrenas F, Palermo RE, Agricola B, Agy MB, Aicher L, Carter V, et al. Deep transcriptional sequencing of mucosal challenge compartment from rhesus macaques acutely infected with simian immunodeficiency virus implicates loss of cell adhesion preceding immune activation. J Virol. 2014;88:7962–72. 10.1128/JVI.00543-14 PMC4097788 24807713

[2] Peng X, Thierry-Mieg J, Thierry-Mieg D, Nishida A, Pipes L, Bozinoski M, et al. Tissue-specific transcriptome sequencing analysis expands the non-human primate reference transcriptome resource (NHPRTR). Nucleic Acids Res. 2015;43:D737–42. 10.1093/nar/gku1110 PMC4383927 25392405

[3] Salinas JL, Kissinger JC, Jones DP, Galinski MR (2014). Metabolomics in the fight against malaria. Mem Inst Oswaldo Cruz.

[4] Joyner C, Barnwell JW, Galinski MR (2015). No more monkeying around: primate malaria model systems are key to understanding Plasmodium vivax liver-stage biology, hypnozoites, and relapses. Front Microbiol.

[5] Hecker M, Lambeck S, Toepfer S, van Someren E, Guthke R (2009). Gene regulatory network inference: data integration in dynamic models-a review. Biosystems.

[6] Styczynski MP, Stephanopoulos G (2005). Overview of computational methods for the inference of gene regulatory networks. Comput Chem Eng.

[7] Zuk O, Margel S, Domany E. On the Number of Samples Needed to Learn the Correct Structure of a Bayesian Network. in *Proceedings of the Twenty-Second Conference on Uncertainty in Artificial Intelligence (UAI2006)* (Cambridge, MA, USA).

[8] Yin W, Kissinger JC, Moreno A, Galinski MR, Styczynski MP. From genome-scale data to models of infectious disease: a Bayesian network-based strategy to drive model development. Math Biosci. 2015 in press. doi:10.1016/j.mbs.2015.06.006 10.1016/j.mbs.2015.06.006 PMC4679518 26093035

[9] Young WC, Raftery AE, Yeung KY (2014). Fast Bayesian inference for gene regulatory networks using ScanBMA. BMC Syst Biol.

[10] Friedman N, Linial M, Nachman I, Pe’er D (2000). Using Bayesian networks to analyze expression data. J Comput Biol.

[11] Tsamardinos I, Brown LE, Aliferis CF (2006). The max-min hill-climbing Bayesian network structure learning algorithm. Mach Learn.

[12] Chickering DM, Heckerman D, Meek C (2004). Large-sample learning of Bayesian networks is NP-hard. J Mach Learn Res.

[13] Kotsiantis SB, Zaharakis ID, Pintelas PE (2006). Machine learning: a review of classification and combining techniques. Artif Intell Rev.

[14] Carvalho AM, Oliveira AL, Sagot MF. Efficient learning of Bayesian network classifiers - An extension to the TAN classifier. *Ai 2007: Advances in Artificial Intelligence, Proceedings*. 2007;4830: 16–25.

[15] Friedman N, Geiger D, Goldszmidt M (1997). Bayesian network classifiers. Mach Learn.

[16] Lin X, Ma P, Li X, Jiang J, Xiao N, Yang F (2012). A learning method of Bayesian network structure. *Fuzzy Systems and Knowledge Discovery (FSKD), 9th International Conference on* 666*–*70.

[17] Cooper GF, Herskovits E (1992). A Bayesian Method for the Induction of Probabilistic Networks from Data. Mach Learn.

[18] Kuschner KW, Malyarenko DI, Cooke WE, Cazares LH, Semmes OJ, Tracy ER (2010). A Bayesian network approach to feature selection in mass spectrometry data. BMC Bioinformatics.

[19] Moreno A, Cabrera-Mora M, Garcia A, Orkin J, Strobert E, Barnwell JW, et al. Plasmodium coatneyi in rhesus macaques replicates the multisystemic dysfunction of severe malaria in humans. Infect Immun. 2013;81:1889–904. 10.1128/IAI.00027-13 PMC3676004 23509137

[20] Lee KJ, Yin W, Arafat D, Tang Y, Uppal K, Tran V, et al. Comparative transcriptomics and metabolomics in a rhesus macaque drug administration study. Front Cell Dev Biol. 2014;2:54. 10.3389/fcell.2014.00054 PMC4233942 25453034

[21] Preininger M, Arafat D, Kim J, Nath AP, Idaghdour Y, Brigham KL, et al. Blood-informative transcripts define nine common axes of peripheral blood gene expression. PLoS Genet. 2013;9:e1003362. 10.1371/journal.pgen.1003362 PMC3597511 23516379

[22] Friedman N, Nachman I, Peer D. Learning Bayesian network structure from massive datasets: The “sparse candidate” algorithm. *Uncertainty in Artificial Intelligence, Proceedings*; 1999. 206–15.

[23] Aliferis CF, Tsamardinos I, Statnikov AR, Brown LE. Causal explorer: A causal probabilistic network learning toolkit for biomedical discovery *Metmbs'03: Proceedings of the International Conference on Mathematics and Engineering Techniques in Medicine and Biological Sciences*; 2003. 371–6.

[24] Abu-Hakmeh KA. Assessing the use of voting methods to improve Bayesian network structure learning. Georgia Institute of Technology: Georgia; 2012.

[25] Spirtes P, Glymour C, Meek C (1993). Causation, Prediction, and Search.

[26] Cheng J, Greiner R, Kelly J, Bell D, Liu WR (2002). Learning Bayesian networks from data: An information-theory based approach. Artif Intell.

[27] Li J, Lenferink AE, Deng Y, Collins C, Cui Q, Purisima EO, et al. Identification of high-quality cancer prognostic markers and metastasis network modules. Nat Commun. 2010;1:34. 10.1038/ncomms1033 PMC2972666 20975711

[28] Steuer R, Kurths J, Daub CO, Weise J, Selbig J (2002). The mutual information: detecting and evaluating dependencies between variables. Bioinformatics.

[29] Boyen X, Friedman N, Koller D. Discovering the hidden structure of complex dynamic systems *Uncertainty in Artificial Intelligence, Proceedings*; 1999. 91–100.

[30] Ghahramani Z (1998). Learning dynamic Bayesian networks. Adaptive Processing of Sequences and Data Structures.

[31] Husmeier D (2003). Sensitivity and specificity of inferring genetic regulatory interactions from microarray experiments with dynamic Bayesian networks. Bioinformatics.

