# Supplementary material for: A tree-like Bayesian structure learning algorithm for small-sample datasets from complex biological model systems
Source: BMC Syst Biol. 2015 Aug 28;9:49. doi: 10.1186/s12918-015-0194-7 (PMC4551520; doi:10.1186/s12918-015-0194-7)
Supplement: Additional file 1: — Supplementary Figures, Tables, and Methods. (DOCX 993 kb) [file 12918_2015_194_MOESM1_ESM.docx]

**Supplementary Figures, Tables, and Methods**

**Figure S1. The underlying structure of the synthetic-tree network.**

**
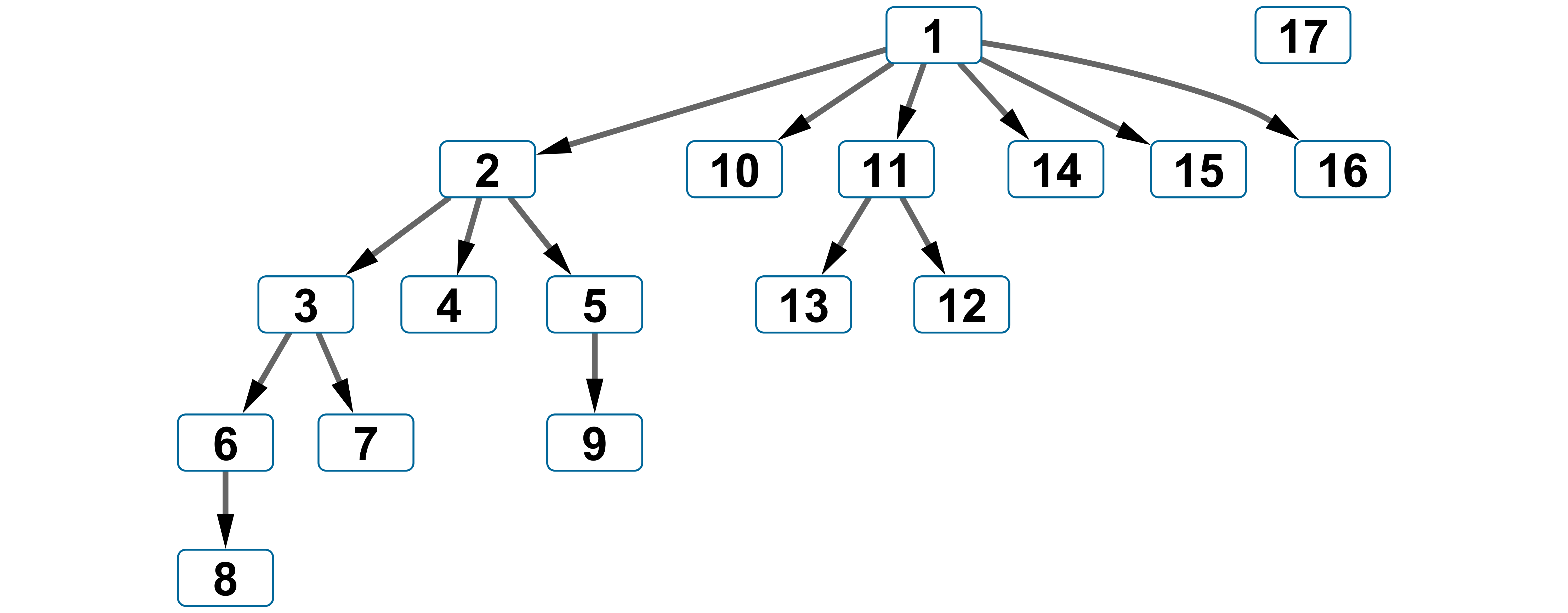
**

**Figure S2. Sensitivity analysis of *ffc* for the Child system with 50 and 200 samples.** Error bars represent one standard deviation. The value of 0.3 used in the main text is not a local optimum. The performance of the algorithm is rather insensitive to small perturbations of ffc, as demonstrated by the fact that variation induced by different datasets is greater than that induced by changing ffc.


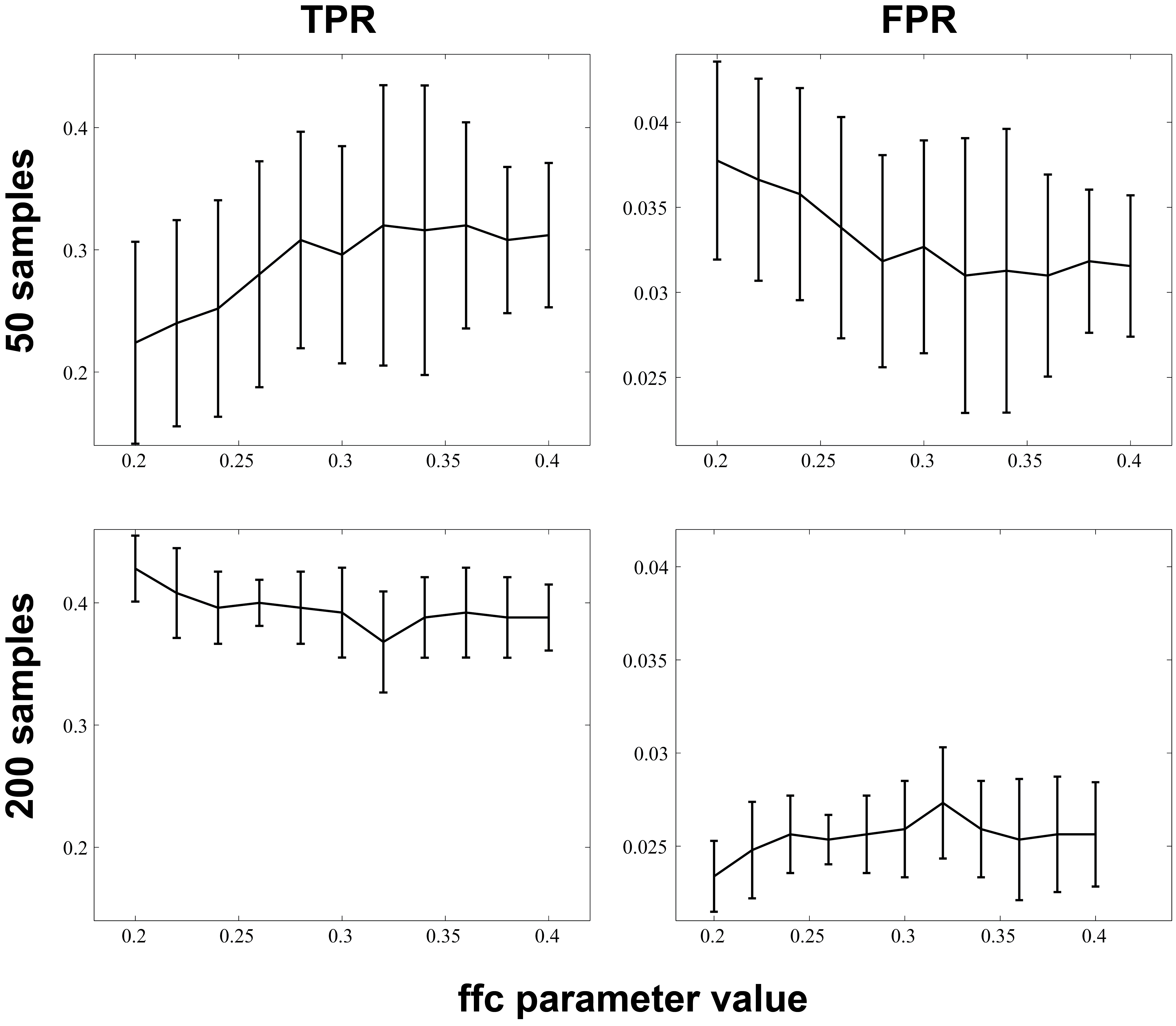


**Figure S3. Sensitivity analysis for SCA method.** All adjustable parameters were varied for SCA using the Child system with a range of sample sizes from 50 to 500. Varying parameters for SCA typically had little effect, suggesting that the results observed for SCA were not due to suboptimal parameter selection. The only slight improvement was in changing the test from mutual information to a Bayesian metric, but even this was insufficient to match the TPR or approach the PPV of the TL-BSLA on the same network. Within each varied parameter (column), different colored lines represent different selections for that parameter. The default value is always the black line, and is constant across each row.

**
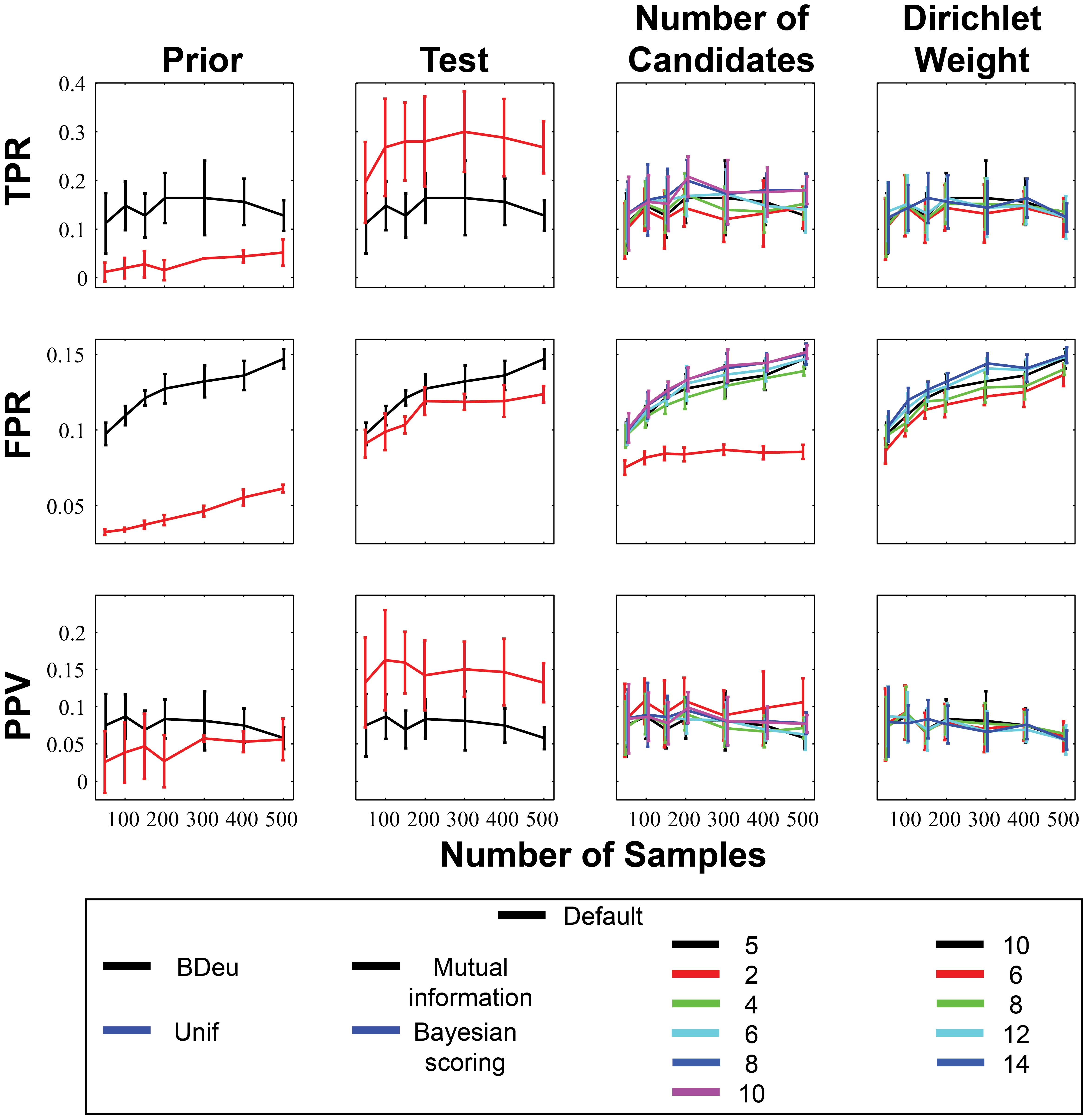
**

**Table S1. Significant features selected by screening procedure for Synthetic tree system.**

| Sample size | Indices of features identified as significant | Fraction of real features selected | Fraction of noisy features selected |
| --- | --- | --- | --- |
| 50 | Real features: 2,3,4,5,6,10,11,12,13,14,15  Noisy features: 20,55,83,91,174 | 69% | 3% |
| 100 | Real features: 2,3,4,5,7,10,11,12,13,14,15  Noisy features: 36,108,117,125,153,161,163,171,174 | 69% | 5% |
| 150 | Real features: 2,3,4,5,6,8,9,10,11,12,13,14,15  Noisy features: 32,38,59,123,130,157 | 81% | 3.5% |
| 200 | Real features: 2,3,4,5,6,7,8,9,10,11,12,13,14,15  Noisy features: 19,21,85,110,139,143,162 | 87% | 4% |
| 300 | Real features: 2,3,4,5,6,7,8,9,10,11,12,13,14,15  Noisy features: 31,52,62,130,173 | 87% | 3% |
| 400 | Real features: 2,3,4,5,6,7,8,9,10,11,12,13,14,15  Noisy features: 26,48,93,126,180 | 87% | 3% |
| 500 | Real features: 2,3,4,5,6,7,8,9,10,11,12,13,14,15  Noisy features: 30,57,76,95,139,154 | 87% | 3.5% |

**Table S2. Significant features selected by screening procedure for Alarm system.**

| Sample size | Indices of features identified as significant | Fraction of real features selected | Fraction of noisy features selected |
| --- | --- | --- | --- |
| 50 | Real features: 1,2,8,15,21,23,25,26,27,28,29,30,31,32,34,36  Noisy features: 38,72,118,238,284,326,335,337,347 | 44% | 2.4% |
| 100 | Real features: 1,2,6,8,10,13,15,20,21,23,25-36  Noisy features: 202,232,253,276,302,328,335,378 | 58% | 2% |
| 150 | Real features: 1,2,6,8,12,15,21,23,25-37  Noisy features: 84,151,157,188,200,272,285,344,382 | 58% | 2.4% |
| 200 | Real features: 1,2,6,7,8,13,14,15,21,24,25-36  Noisy features: 61,91,102,198,236,272,330,337,349 | 61% | 2.4% |
| 300 | Real features: 1,2,6,8,12,13,15,16,21,23,25-37  Noisy features: 48,162,219,222,240,300 | 64% | 1.7% |
| 400 | Real features: 1,2,4,6,8,13,15,21,23,25-37  Noisy features: 38,127,256,296 | 61% | 1% |
| 500 | Real features: 1,2,5,6,8,13,15,21,23,25-37  Noisy features: 44,94,199,205,239,302,306,312,358 | 61% | 2.4% |

**Supplementary methods**

The experimental protocol was similar to that used in our previous malaria challenge experiment^1^, with four noteworthy exceptions: there was a longer follow-up period for measurements, complete blood count (CBC) profiles were determined every day, there was no biotinylation of erythrocytes, and *Plasmodium cynomolgi* sporozoites were used for the experimental infection. Briefly, five male macaques were injected intravenously with a preparation of approximately 2,000 freshly isolated *P. cynomolgi* B strain sporozoites from *Anopheles dirus* salivary glands. The sporozoites were generated by allowing laboratory-bred *Anopheles dirus* mosquitoes to feed on a donor rhesus macaque after gametocytes were observed in the blood. That donor macaque was inoculated at the Centers for Disease Control and Prevention (Atlanta, GA, USA) with blood-stage parasites from cryopreserved stocks. Before resuspension and injection, the isolated sporozoites were washed to remove microorganisms found in salivary glands. Eighty microliters of blood was collected in EDTA-coated tubes daily for CBC profiles and counting of parasites. Animals that developed clinical complications due to severe malaria received a subcurative treatment of 1 mg/kg of artemether. At IACUC-approved infection milestones, all animals received a blood stage curative treatment of 4 mg/kg once followed by 2 mg/kg/day for one week.

Bone marrow aspirates were taken under anesthesia with ketamine at seven time points over the course of approximately 100 days, corresponding to baseline, peak of parasitemia, treatment of blood-stage parasites, and during and after relapse. Transcriptional profiles were obtained by sequencing on an Illumina HiSeq2000 at the Yerkes National Primate Research Center Genomics Core. Reads were mapped to a recent version of the macaque genome and, after quality control and normalization steps, differential expression of genes was determined using DESeq software^2^. The supervised normalization of microarrays procedure^3^ (SNM) in R was used to remove animal effects from the data due to significant baseline differences between the animals. The resulting animal-adjusted transcriptional data was used for input for the TL-BSLA algorithm.

**Supplementary References**

1. Moreno A., Cabrera-Mora M., Garcia A., Orkin J., Strobert E., Barnwell J. W. and Galinski M. R. Plasmodium coatneyi in rhesus macaques replicates the multisystemic dysfunction of severe malaria in humans. *Infect Immun* **81**: 1889-904, 2013.

2. Anders S. and Huber W. Differential expression analysis for sequence count data. *Genome Biol* **11**: R106, 2010.

3. Mecham B. H., Nelson P. S. and Storey J. D. Supervised normalization of microarrays. *Bioinformatics* **26**: 1308-15, 2010.
